# Insufficient maintenance DNA methylation is associated with abnormal embryonic development

**DOI:** 10.1186/1741-7015-10-26

**Published:** 2012-03-13

**Authors:** Li-Jun Yin, Yu Zhang, Ping-Ping Lv, Wei-Hua He, Yan-Ting Wu, Ai-Xia Liu, Guo-Lian Ding, Min-Yue Dong, Fan Qu, Chen-Ming Xu, Xiao-Ming Zhu, He-Feng Huang

**Affiliations:** 1 Department of Reproductive Endocrinology, Women's Hospital, School of Medicine, Zhejiang University, 1 Xueshi Road, Hangzhou, Zhejiang 310006, China; 2 Key Laboratory of Reproductive Genetics, Ministry of Education, 1 Xueshi Road, Hangzhou, Zhejiang 310006, China; 3 Department of Obstetrics and Gynecology, The First Affiliated Hospital, School of Medicine, Zhejiang University, 79 Qingchun Road, Hangzhou, Zhejiang 310003, China

## Abstract

**Background:**

Early pregnancy loss (EPL) is a frustrating clinical problem, whose mechanisms are not completely understood. DNA methylation, which includes maintenance methylation and *de novo *methylation directed by DNA methyltransferases (DNMTs), is important for embryo development. Abnormal function of these DNMTs may have serious consequences for embryonic development.

**Methods:**

To evaluate the possible involvement of DNA methylation in human EPL, the expression of DNMT proteins and global methylation of DNA were assessed in villous or decidua from EPL patients. The association of maintenance methylation with embryo implantation and development was also examined.

**Results:**

We found that DNMT1 and DNMT3A were both expressed in normal human villous and decidua. DNMT1 expression and DNA global methylation levels were significantly down-regulated in villous of EPL. DNMT3A expression was not significantly changed in the EPL group compared to controls in either villous or decidua. We also found that disturbance of maintenance methylation with a DNMT1 inhibitor may result in a decreased global DNA methylation level and impaired embryonic development in the mouse model, and inhibit *in vitro *embryo attachment to endometrial cells.

**Conclusions:**

Our results demonstrate that defects in DNA maintenance methylation in the embryo, not in the mother, are associated with abnormal embryonic implantation and development. The findings of the current study provide new insights into the etiology of EPL.

## Background

Early pregnancy loss (EPL) is one of the most common reproductive failures of human pregnancy. It is characterized as high incidence, but the etiology is ambiguous. At least 75% of women experience one or more EPL, which includes loss of unrecognized or histologically recognized pregnancies. The incidence of recognized EPL is approximately 10% to 15% [1]. Although there are various possible etiological factors associated with EPL, such as embryonic chromosomal abnormality, maternal endocrine diseases, anatomical abnormalities of the reproductive system, environmental influences and immunologic factors, the molecular mechanisms of EPL remain incompletely described [2-4].

In mammals, the global DNA epigenetic profile of the genome is dynamically reprogrammed during embryogenesis and the early development of the fetus [5]. These dynamic processes are vital for the fertilization and histological differentiation of the embryo [6]. DNA methylation, as the primary regulator of hereditary information, occurs exclusively at CpG dinucleotides [7]. These CpG islands are often among the promoter regions of genes and methylation of CpG islands results in transcriptional repression However, aberrant DNA methylation has been found to lead to abnormal embryonic development, birth malformations and other diseases, such as human carcinoma [8,9].

*De novo *methylation and maintenance methylation are distinct processes that are required for the establishment and inheritance of tissue-specific methylation patterns, especially during implantation. Three main DNA methyltransferase enzymes (DNMT) including DNMT1, DNMT3A and DNMT3B have been characterized [10]. DNMT1, with its high affinity for hemimethylated DNA *in vitro*, predominantly catalyzes maintenance methylation via binding to proliferating cell nuclear antigen in replication foci during S phase [11]. DNMT3A and DNMT3B are mainly responsible for *de novo *methylation that establishes a new DNA methylation state at repeat sequences, imprinted genes, and developmental genes [12,13].

The establishment of appropriate methylation patterns depends on a methodical regulation of the methyltransferase activity. Dysfunction of any of these DNMTs may have serious consequences for embryonic development and subsequent gestation, even embryonic lethality, as the epigenetic status of genes or repeat sequences have a profound effect on cell physiology and can markedly alter embryonic and fetal development [12,14-16]. It was reported that DNMT1^-/- ^mice embryos have genome-wide demethylation and developmental arrest at the early stage of gestation [17]. DNMT3a^-/- ^or DNMT3b^-/- ^mice failed to initiate *de novo *methylation after implantation and also exhibited embryonic development arrest [14,18]. Inefficient epigenetic reprogramming caused by abnormal expression of DNMTs may be closely associated with a high rate of abortion and developmental abnormalities in bovine clones [19]. However, it remains unclear whether dysfunction of DNMTs is involved in human early pregnancy loss.

To evaluate the role of DNA methylation in the pathogenesis of early pregnancy loss, we detected the expression of DNMTs protein and global DNA methylation state in human EPL and found that insufficient maintenance methylation existed in the villi of EPL. Further, we found that inhibition of DNMT1 impaired embryo implantation and embryonic development capacity *in vivo*. The findings of the current study indicated that defects in maintenance methylation in the embryo were associated with abnormal embryonic implantation and development.

## Methods

### Human subjects and sample collection

Villous and decidual tissues were collected from patients with EPL and women undergoing selective pregnancy termination in the first-trimester (gestational age, seven to nine weeks) for nonmedical reasons in Women's Hospital, School of Medicine, Zhejiang University, between September 2006 and May 2007. Tissues were snap-frozen in liquid nitrogen and stored at -80°C until assay by Western blot analysis or fixed in paraformaldehyde (PFA) for immunohistochemistry. EPL was diagnosed based on the presence of vaginal bleeding and/or lower abdominal pain, together with the findings of pelvic and ultrasonic examination. Exclusion criteria included chromosomal abnormalities, endocrine diseases, infections and anatomical abnormalities of the genital tract, immunological diseases, trauma, signs of other concurrent medical complications and any chemical agent intake before sample collection [20]. Written informed consents were obtained from all patients who provided samples and the protocol was approved by the Ethical Review Committee of Women's Hospital, School of Medicine, Zhejiang University.

### Collection of mouse embryos and tissues

Animal care followed the guidelines recommended by the Animal Care and Use Committee (ACUC) of the School of Medicine, Zhejiang University. Female ICR mice (six to seven weeks old) were synchronized and superovulated by intraperitoneal injection of 7.5 IU pregnant mare serum gonadotropin (PMSG) and human chorionic gonadotropin (hCG, Organon, Oss, The Netherlands). Thirty mice were bred with proven breeder males and were checked the next day for a copulation plug (designated as Day 0 of gestation). Twenty pregnant mice were sacrificed on day 4 to collect blastulas by flushing the uterus with modified human tubal fluid (MHTF; Irvine Scientific, Irvine, CA, USA) as described previously [21]. The pregnant mice were anesthetized by 10% chloral hydrate (0.4 ml/100 g, Sinopharm Chemical Reagent, Beijing, China) on the fifth day of gestation (around implantation time) and then subjected to operation on the loin to expose the uterine horns. Five microliters of procainamide (1 mM) (Sigma, St. Louis, MO, USA) was injected slowly into the left side uterine cavity using a 30-gauge needle and 5 μl sterile saline (vehicle) was injected into the right side as control. The incision was then closed and the mice were sacrificed on day 11 to observe the number of embryos implanted and embryonic development. Mouse embryos were collected and stored at -80°C for assay of global DNA methylation. Uteri were collected and then either fixed in PFA for immunohistochemistry analysis or Haematoxylin & Eosin (H&E) staining.

### Cell culture and embryo attachment test

Ishikawa cells were cultured in Ham F-12/(D)MEM (1:1) containing 30% fetal calf serum, progesterone (63.5 nmol/L), estradiol-17β (7.14 nmol/L), insulin (100 mg/ml) and epidermal growth factor (20 ng/ml) in a humidified condition composed of 20% oxygen and 5% carbon dioxide at 37°C.

Embryos (n = 142) obtained via flushing mouse uteri were rinsed three times in MHTF and cultured in the presence or absence of procainamide (0.5 mM), a DNMT1 inhibitor, for 24 hours. The embryos were then transferred to a dish covered with a single layer of Ishikawa cells that mimics endometrial cells as described previously [22]. The number of implanted embryos was counted at 48 hours and 72 hours of culture. The presence of blastocyst adherence to the dish bottom and the erosion of Ishikawa cells by the blastocyst invasion were considered successful embryo implantation.

### Western blotting analysis

Samples of 150 mg villous or decidual tissues were homogenized in 500 μl of ice-cold lysis buffer containing 50 mM Tris-HCl (pH 8.0), 150 mM NaCl, 0.02% sodium azide, 1% Nonidet P-40 (NP-40), 0.5% sodium deoxycholate, 100 μg/ml phenylmethyl-sulfonyl fluoride (PMSF) (Sigma), and 100 μg/ml leupeptin (Sigma). The supernatant was collected after centrifugation at 15,000 g for 20 minutes at 4°C and protein concentrations were determined using the Bradford method (Bio-Rad Laboratories, Hercules, CA, USA).

Western blot analysis of DNMT1 and DNMT3A was performed as previously described [19]. Briefly, the proteins (60 μg) were separated on SDS-PAGE and probed by anti-DNMT1 (Novus Biologicals, Littleton, CO, USA) or anti-DNMT3A (Santa Cruz Biotechnology, Santa Cruz, CA, USA) and anti-β-actin antibody (Santa Cruz Biotechnology) overnight at 4°C. Then the membranes were incubated with second antibody for 1 hour at room temperature after thorough washing with buffer. The signal was visualized with ECL kits according to the manufacturer's instructions (Amersham Biosciences, Little Chalfont, Buckinghamshire, UK) and exposed to film. The density of immunoblotting was quantified with the software Quantity One (Bio-Rad Laboratories, Hercules, CA, USA). The expression of target proteins was normalized against internal control β-actin.

### Immunohistochemistry and immunocytochemistry analysis

Tissues fixed in PFA were dehydrated and embedded in paraffin. Sections of 4 micrometers were cut and mounted onto slides. After deparaffinization, rehydration and blockade, sections were rinsed three times with washing buffer, incubated with primary antibody (rabbit anti-DNMT1 (Novus Biologicals, Littleton, CO, USA)) and rabbit anti-DNMT 3A (Santa Cruz Biotechnology) for 1 hour at room temperature. After rinsing three times with washing buffer, the sections were incubated with horseradish peroxidase conjugated goat anti-rabbit IgG (Santa Cruz Biotechnology) for 30 minutes. The signals were visualized with DAB+ Substrate Chromogen solution (DAKO, Tokyo, Japan) and observed under Eclipse E400 microscopy (Nikon, Tokyo, Japan).

To detect the expression of leukemia inhibitory factor (LIF) in Ishikawa cells, cells were mounted to coverslips, fixed with PFA and permeated with Triton X-100. Rabbit anti-LIF antibody (1:50 dilution, Santa Cruz Biotechnology) was used as the primary antibody for the detection of LIF. The signal was visualized as described above.

### Global methylation of DNA in human villous and mouse embryo

Genomic DNA was extracted from human villous of the EPL group and the normal pregnant group and mouse embryos at day 11 with a commercially available kit (Axygen, Union City, CA, USA). The level of global DNA methylation was determined by the Methylamp Global DNA Methylation Quantification Ultra Kit (Epigentek, Farmingdale, NY, USA). In this assay, 5-methylcytosine-modified genomic DNA is recognized by 5-methylcytosine antibody and the bound DNA is quantified in a colorimetric reaction. Positive (methylated) and negative (unmethylated) control DNA was supplied with the kit. The proportion of methylated nucleotides in the total genomic DNA was shown (methylation %). For sample calculation of DNA methylation, the following formula was used: Methylation % = (Sample OD-Negative Control OD)/X*/(Positive Control OD-Negative Control OD) × 10 × 100% (* × is the GC content of the species DNA (GC content is 41% for human genomic DNA, 42% for mouse). All procedures were performed according to the Kit's User Guide. (http://www.epigentek.com/catalog/redirect.php?action=url&goto=www.epigentek.com%2Fdocs%2FP-1014B.pdf).

### Statistics

The expression of DNMT1 and DNMT3A and the level of global DNA methylation are presented in means ± SEM and compared with the Mann-Whitney test. Chi-square test was used to compare the rate of *in vitro *embryonic implantation and the paired *t*-test was used to test the *in vivo *effect of DNMT1 inhibition on embryo implantation. Animal experiments were replicated at least three times. *P <*0.05 was considered statistically significant. Statistical analysis was performed using the SPSS 16.0.

## Results

### Decreased DNMT1 expression level in human villous of EPL

**Figure 1:**
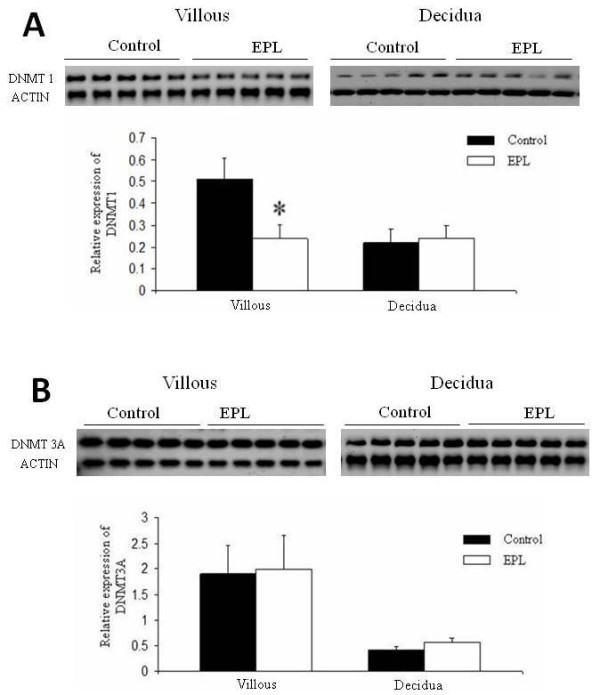
**Alterations of expression levels of DNMT1 and DNMT3A in human villous and deciduas detected by western blot**. **A**: The expression level of DNMT1 in villous was significantly lower in the EPL group than in the control. There was no difference in the DNMT1 expression level in the decidua between the two groups. **B**: The expression level of DNMT3A was not significantly different in either the villous or decidua between the EPL and control groups. The data are shown as means ± SEM. EPL group: n = 16; control group: n = 16. * *P *< 0.05. EPL, early pregnancy loss; SEM, standard error of the mean.

**Figure 2:**
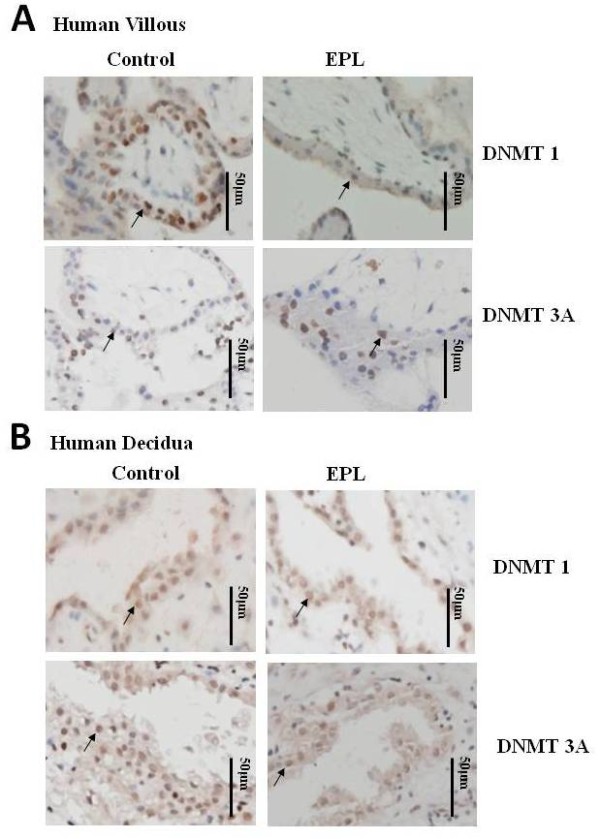
**Immunohistological detection of DNMT1 and DNMT3A in human villous and decidua**. **A**: The staining of DNMT1 and DNMT3A were both localized in the nucleus of villous trophoblasts. **B**: The staining of both DNMT1 and DNMT3A was localized in the nucleus of the glandular epithelium of decidua (The bar represents 50 μm).

Western blot analysis revealed that the DNMT1 expression level was significantly lower in villous of women with an EPL (n = 16) than controls (n = 16) (*P *= 0.023, Figure 1A), but there was no significant difference in DNMT1 expression in the decidua (*P *= 0.815, Figure 1A). There were no significant differences in the expression level of DNMT3A in either villous or decidua between EPL (n = 16) and control (n = 16) specimens (*P *= 0.294 and P = 0.194, respectively, Figure 1B). Immunohistochemistry analysis showed the localization of both DNMT1 and DNMT3A in the nucleus of trophoblast and the nucleus of the glandular epithelium of decidua (Figure 2).

### Reduced DNA methylation level in villous of EPL

**Figure 3:**
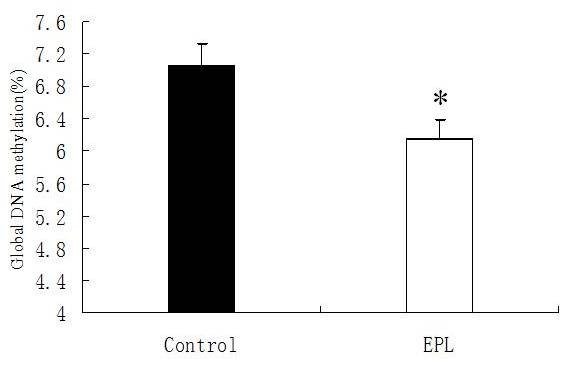
**Alterations of global DNA methylation level in villous**. The global DNA methylation level was significantly down-regulated in human villous of the EPL group compared to the control group. The data are shown as means ± SEM. EPL group: n = 16; control group: n = 16. * *P *< 0.05. EPL, early pregnancy loss; SEM, standard error of the mean.

The levels of global DNA methylation in the villous of women with EPL (n = 16) and normal pregnant women (n = 16) are shown in Figure 3. The global DNA methylation was significantly reduced in villous from women with EPL compared to controls (*P *= 0.0164). It was noticed that these assays are influenced strongly by mCpG density, such that regions that are more highly methylated are detected with much greater ease than regions of low methylation density. Thus, we did not observe the absolute level of DNA methylation, but showed a difference between the groups being compared.

### Inhibition of DNMT1 activity impaired *in vivo *embryo implantation and development

**Figure 4:**
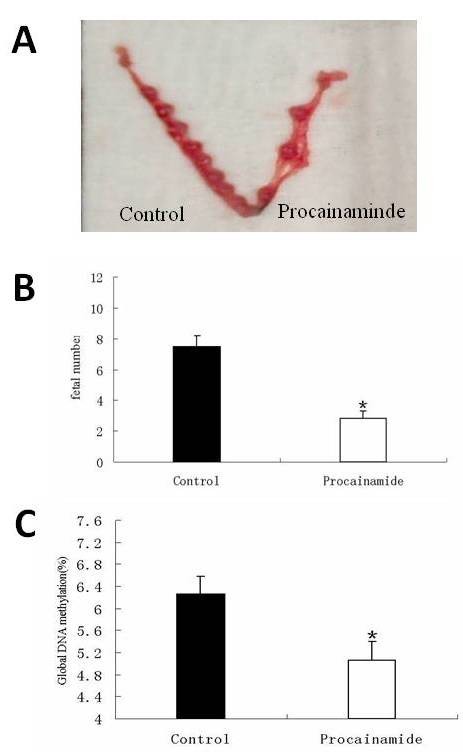
**The effect of DNMT1 inhibition on embryo implantation and DNA methylation level *in vivo***. **A**: The fetal number was significantly less in the uterine horn injected with DNMT1 inhibitor than in the control horn. The data are shown as means ± SEM. Procainamide group: n = 10; control group: n = 10. * *P *< 0.05. **B**: The global DNA methylation level was significantly lower in mouse embryos obtained from the uterine horn injected with DNMT1 inhibitor than in those from the control horn. The data are shown as means ± SEM. Procainamide group: n = 18; control group: n = 18. * *P *< 0.05. SEM, standard error of the mean.

**Figure 5:**
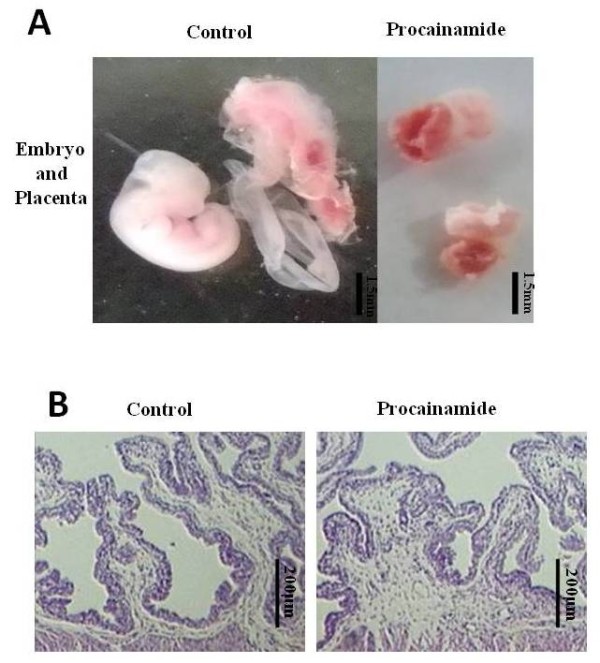
**The effects of DNMT1 inhibition on embryonic and placental development**. **A**: The embryos and placenta developed normally in the control horn but the embryos and placenta in the uterine horn injected with a DNMT1 inhibitor showed growth retardation (The bar represents 1.5 mm). **B**: H & E staining showed that there was no obvious structural difference in the endometrial tissue of the non-implantation site between the two sides of uterine horn with or without DNMT1 inhibitor injection (The bar represents 200 μm). H & E, hematoxylin and eosin.

Fifty percent (n = 10) of mice injected with procainamide, an inhibitor of DNMT1, in the uterus presented with vaginal bleeding but recovered before Day 11 of gestation. On Day 11, the number of embryos implanted in the uterine horn injected with procainamide was significantly reduced compared with controls (n = 10), *P *< 0.05 (Figure 4A and 4B). The global DNA methylation level of embryos obtained from the procainamide-injected horn was significantly lower than that of controls (*P *= 0.0155, Figure 4C). The mouse embryos and placentas obtained from the control horn appeared to be normal, but those from the procainamide-injected horn were abnormally developed (Figure 5A). H & E staining of the mouse uterus of non-implantation sites revealed a similar structure between procainamide-injected horns and control horns (Figure 5B).

### Blockade of DNMT1 activity inhibited embryo attachment to endometrial cells

**Figure 6:**
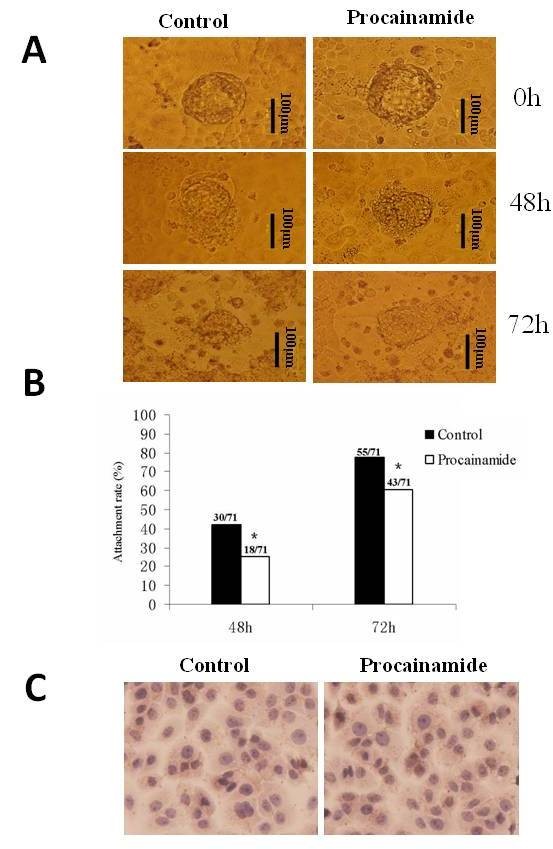
**The effect of DNMT1 inhibition on embryonic attachment *in vitro***. **A**: Blastulas collected from ICR mice were pre-treated with or without DNMT1 inhibitor and placed on an Ishikawa cell-covered plate. Erosion of Ishikawa cells occurred after implantation (The bar represents 100 μm). **B**: Pretreatment with DNMT1 inhibitor significantly decreased the implantation rate of embryos *in vitro*. The rate of embryo attachment to Ishikawa cells was 30/71 (42%) and 55/71 (77%) at 48 hours and 72 hours, respectively, for the control group. The rate decreased to 18/71 (25%) and 43/71 (61%) at 48 hours and 72 hours, respectively, in the procainamide treatment group (* *P *< 0.05). **C**: The presence of DNMT1 inhibitor did not affect the LIF expression in Ishikawa cells. LIF, leukemia inhibitory factor.

The number of embryos attached to Ishikawa cells was 30/71 (42%) and 55/71 (77%) at 48 hours and 72 hours, respectively. The inhibition of DNMT1 with procainamide significantly decreased the embryo attachment rate to 18/71 (25%) and 43/71 (61%) at 48 hours and 72 hours, respectively (*P *= 0.033 for 48 hours; *P *= 0.029 for 72 hours) (Figure 6). We found that inhibition of DNMT1 activity did not significantly affect the expression of LIF, a marker of endometrial receptivity, in Ishikawa cells (Figure 6).

## Discussion

During mammalian embryo implantation and development, DNA methylation undergoes dramatic reprogramming that is crucial for the development of both the embryo and the maternal endometrium [23-26]. In the present study, we showed that DNMT1 and DNMT3A were expressed in human villous trophoblast and glandular epithelial cells of decidua. DNMT1 was significantly down-regulated in the villous of women with EPL, but not in the decidua. DNMT3A expression was not significantly changed in the EPL group compared to the control group. The global DNA methylation level was significantly lower in the EPL villous than in villous from the controls. These findings suggest that insufficient embryonic maintenance methylation is associated with abnormal embryonic development in human early pregnancy loss. Evidence from *in vitro *and *in vivo *studies confirmed the importance of maintenance DNA methylation in embryo implantation and development. To the best of our knowledge, this is the first report describing defects in DNA maintenance methylation in the pathogenesis of EPL.

In the present study, we first demonstrated that DNMT1 and DNMT3A proteins were expressed in the nuclei of the villous trophoblast and glandular epithelium of normal human decidua. DNMT3B, a *de novo *methyltransferase, was detected with low expression in decidual tissue, but not in villous (data not shown). The most likely explanation is that DNMT3B is not expressed in extra-embryonic lineages of post-implantation embryos [27]. The expression of both DNA methyltransferases suggests that DNMT1 and DNMT3A may be DNA methylation regulatory enzymes at the feto-maternal interface during early embryo development. Subsequent semiquantitative analysis supported a pathological role of DNMT1 in EPL. *De novo *methyltransferase DNMT3A expression had no evident relationship with EPL. A possible reason might be that *de novo *methylation mainly happens during epigenetic reprogramming in the gametes and preimplantation embryo [28,29]. After embryo implantation, the methylation is maintained by DNMT1 [30] and there is no noticeable *de novo *methylation change in the post-differentiated human villous [31].

For the global methylation assay, we used the Methylamp™ Global DNA Methylation Quantification Ultra Kit. This commercial product for global methylation assay is commonly used [32]. The assay uses a 5-methylcytosine antibody to distinguish methylated from unmethylated cytosine. These assays are influenced significantly by mCpG density so that more highly methylated regions are detected better than regions of low methylation density. These methods do not offer accurate quantitative results; however, they do allow comparisons between groups. The methylation levels in the EPL group and the control group were measured at the same time with the same kit to decide whether there is a relative difference of DNA methylation level between the two groups. Our results show that, compared with the control group, global DNA methylation was significantly reduced in the EPL group both in the patient samples and in the animal model.

The alterations in the expression of DNMT1 and global demethylation in villous of the EPL women suggest that aberrant maintenance DNA methylation may be involved in the pathogenesis of human EPL. It was reported that mouse embryos lacking Dnmt1 showed genome-wide demethylation and knockout of Dnmt1 in embryos led to a complete loss of methylation at both paternally and maternally methylated differential methylation regions (DMRs) [17]. Mice deficient in the maintenance of genomic imprints exhibited significant developmental delays in multiple organ systems [33]. Since demethylation of the genome decreases cell proliferation [15] and the aborted villous has less cell proliferation and more cell apoptosis than normal villous [34], insufficient DNA methylation may influence cell proliferation, migration and invasion, thus affecting embryonic implantation and post-implantation development [35].

Implantation is the first and crucial step in governing reproductive outcomes [36]. Fundamental to this process are dynamic and precisely ordered molecular and cellular events, driven by the combination of embryo and host-receptive-endometrium to facilitate the establishment of the maternal-fetal interface. Regulation of DNA methylation plays an important role in this process [35]. In our *in vitro *experiment of embryo implantation, the reduction of the implantation rate may also be caused by abnormal trophoblastic cell proliferation, migration and invasion. In addition, the expression of LIF by Ishikawa cells in the presence of a DNA methylation inhibitor did not significantly change, testifying to the acceptance of our embryonic adhesion/implantation model. LIF is one of the crucial cytokines in the endometrium or at the maternal-fetal interface [37]. The steady expression of LIF in the presence of a DNA methylation inhibitor suggests that the disturbance of maintenance methylation may not influence the endometrial receptivity for embryos.

The findings of our mouse experiment confirm that disturbance of maintenance DNA methylation plays an important role in the pathogenesis of EPL. Female mice that received a uterine injection of a DNA methylation inhibitor presented typical symptoms of EPL with low implantation and pregnancy rates, and the embryos in the treated uterine horn showed global DNA demethylation. The number of fetuses in the treated uterine horn was less than the control horn, and the aborted fetuses and placentas in the treated horn had incomplete development of organs and obscured tissue structures. It has been shown that knockdown of DNMT1 results in decreased cell viability [38]. Further research indicated that DNMT1 had an intimate relationship with DNA replication and the cell cycle, since inhibition of DNA methyltransferase interferes with DNA replication, and DNMT1 depletion triggers intra-S-phase cell cycle arrest [39]. In the realm of organism development, DNA methylation also has long-term effects on the development of the fetus and placenta. Non-specific demethylation reagents have been found to cause growth retardation, malformation, fetal lethality and abnormal tissue structure in the placenta [40]. Thus, we speculated that the poor formation of the embryonic organs and tissues in our study might be induced by the demethylation of the promoters of important developmental genes, thereby resulting in poor conditions for pregnancy. However, a thorough study of the mechanisms underlying the effect of DNA demethylation on early pregnancy loss is needed.

## Conclusions

In summary, defects in DNA methylation maintenance were demonstrated in the villous of human EPL tissues. Inhibition of DNA methylation maintenance led to a decreased implantation rate of embryos, increased fetal absorption, and poor fetal and placental development. These observations suggest that the embryonic defect in DNA methylation maintenance may be associated with abnormal embryonic implantation and development. Our findings provide new insights into the etiology of unexplained EPL. However, further experiments are needed to clarify changes in gene expression and methylation states of genes under the DNA methylation maintenance defect in EPL pathogenesis.

## Abbreviations

DMRs: differential methylation regions; DNMTs: DNA methyltransferases; EPL: early pregnancy loss; H&E: Haematoxylin & Eosin; LIF: leukemia inhibitory factor; MHTF: modified human tubal fluid; PFA: paraformaldehyde; PMSF: phenylmethyl-sulfonyl fluoride; PMSG: pregnant mare serum gonadotropin.

## Competing interests

The authors declare that they have no competing interests.

## Authors' contributions

LJY and YZ participated, together with HFH, in the design of the study. LJY, YZ, PPL, WHH, YTW, AXL and GLD carried out the experiments. Data analysis was performed by LJY, YZ, FQ and CMX. The manuscript was written by LJY, YZ, MYD and XMZ. HFH critically read the manuscript. All authors read and approved the final manuscript.

## Pre-publication history

The pre-publication history for this paper can be accessed here:

http://www.biomedcentral.com/1741-7015/10/26/prepub
